# Quantitative Risk Assessment of African Swine Fever Introduction into Spain by Legal Import of Live Pigs

**DOI:** 10.3390/pathogens11010076

**Published:** 2022-01-08

**Authors:** Carolina Muñoz-Pérez, Jaime Bosch, Satoshi Ito, Marta Martínez-Avilés, José Manuel Sánchez-Vizcaíno

**Affiliations:** 1 VISAVET Health Surveillance Centre, Animal Health Department, Complutense University of Madrid, 28040 Madrid, Spain; jbosch@ucm.es (J.B.); satosito@ucm.es (S.I.); jmvizcaino@ucm.es (J.M.S.-V.); 2 Animal Health Research Centre (CISA-INIA/CSIC), 28130 Madrid, Spain; mmaviles@ucm.es

**Keywords:** African swine fever, import, live pig, quantitative risk assessment, Spain

## Abstract

African swine fever (ASF) is a devastating infectious disease of pigs that is threatening the global swine industry at present. The current spread of ASF in Europe and its recent incursion into Germany pose a serious risk to Spain, one of the world’s leading pig producers. A quantitative stochastic risk assessment model was developed to estimate the probability of ASF introduction into Spain via the legal import of live pigs. The results suggest a low annual probability of ASF introduction into Spain (1.07 × 10^−4^), the highest risk being concentrated in Central European countries (Germany, the Netherlands, Belgium, and Luxembourg) during the months of April and February. The methods and results presented herein could contribute to improving prevention and control strategies and, ultimately, would help reduce the risk of ASF introduction into Spain.

## 1. Introduction

African swine fever (ASF) is one of the most devastating diseases of swine. It is listed as notifiable by the World Organisation for Animal Health (OIE) due to its high mortality, the lack of an effective vaccine or treatment, and the serious economic consequences. ASF is a haemorrhagic disease caused by African swine fever virus (ASFV), the only member of the *Asfarviridae* family [1]. The disease affects all members of the family *Suidae*, of all ages and genders, and can have different clinical presentations varying from a peracute form, with a mortality rate of 100%, to a chronic form [2]. ASF was first reported in 1921 in Kenya [3], and since then it has been reported in many African countries. The disease was confined to Africa until 1957, with an outbreak in Portugal, which was rapidly controlled. Again in 1960, the disease was reported in Portugal and spreading to the entire Iberian Peninsula, remaining endemic for 35 years, with sporadic outbreaks in other European and American countries [4]. In 2007, the disease reached the European continent again, with an outbreak in Georgia, and since then it has spread worldwide despite intensive control efforts [5]. Nowadays, ASF affects more than 55 countries on five continents (Africa, America, Asia, Europe, Oceania), including some of the world´s largest exporters of pig and pig products, such as China and Germany, who have been affected since August 2018 and September 2020, respectively [5].

Although the disease has several transmission paths, one of the most important factors for this widespread diffusion across great distances is the vast amount of international trade of live swine and their products [6]. The import of live animals, specifically trucks returning from infected to uninfected areas, is considered one of the most important risk factors for the introduction of contagious animal diseases into an ASF-free country [7,8]. Indeed, authors concluded that the risk of ASF introduction into the EU was mainly associated with returning trucks that were not properly disinfected [9].

To prevent or reduce the risk of ASF introduction, the World Trade Organization (WTO) and the World Organization for Animal Health (OIE) recommend preventive and control measures. Among others, the trade of live swine and their products from ASF-affected countries or zones to ASF-free countries or zones is not allowed [10]. In the light of these policies, the greatest risk for ASFV introduction by legal trade would be concentrated from the time of initial infection until it is detected and reported to the OIE, a period known as the “high-risk period” [11]. 

The present study quantifies the risk of ASF introduction into Spain through the legal import of live pigs from 2012 to 2020. The analysis aimed to identify geographical areas and time periods that pose the highest risk for ASFV introduction into Spain and, ultimately, to inform and reinforce control and prevention strategies as required. 

## 2. Material and Methods

### 2.1. Definition of the Unit of Analysis and Data Sources

The potential risk of the introduction of ASF into Spain by the import of live animals varies depending on the country of origin of the pigs. Likewise, the amount of trade varies throughout the year. Therefore, the risk of ASF introduction was quantified per month and per country of origin.

Data regarding the number of live pigs imported by Spain from the origin countries per month from 2012 to 2020 were obtained from the Statistical Office of the European Union (EUROSTAT database) [12]. The assessment considered 13 origin countries for which data are available. 

This model updates, in the case of Spain, the results obtained for the risk estimation for the introduction of ASF into the European Union by the legal import of live pigs [13]. Table 1 summarizes all assumptions, input parameters, parameterization, and sources of information used in the quantitative model.

### 2.2. Model Formulation

The probability of ASF introduction into Spain by the legal import of live swine (PI) was estimated per month (m) and per country of origin of imported pigs (c), assuming a binomial process of the form:

PI = Σ1 − (1 − p_cm_) ^ncm^ (1)

where _ncm_ is the number of live pigs imported from country of origin (c) during the month (m), and p_cm_ is the conditional probability that an infected pig was introduced from country (c) during the month (m) and effectively contacted a susceptible pig.

**Figure 1:**
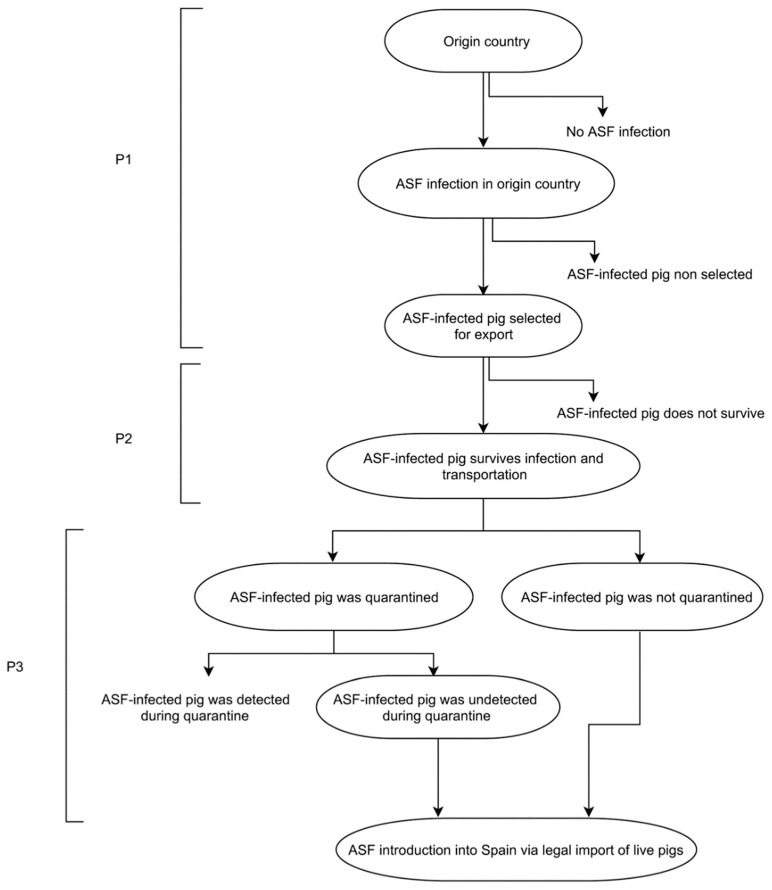
Event tree of ASF introduction into Spain via legal import of live pigs.

The conditional probability (p_cm_) was estimated as the product of three independent events. The probability of selecting an infected pig from country c before detection of ASFV infection (P_1_); the probability that the infected pig survives and reaches the holding (P_2_); and the probability that the imported pig has effective contact with at least one susceptible animal in the holding, resulting in disease transmission (P_3_) (see Figure 1). 

The model was developed using @Risk version 8.2. (Palisade Corporation, Newfield, NY, USA) on Microsoft Excel 2013® and run for 10,000 iterations using the Monte-Carlo sampling method. Map to visualize risk distribution was generated in ArcGis 10.4.1 (ESRI, Redlands, CA, USA).

### 2.3. Definition of Input Parameters

#### 2.3.1. Probability of Selecting an Infected Pig (P_1_)

The probability of exporting an infected animal from country *c* at month *m* to Spain before detection of ASF was modelled using a beta distribution with parameters α_1p_ and α_2p_. These parameters were calculated as:

α_1p_ = NI + 1 and α_2p_ = No − (NI+1) (2)

where NI is the estimated number of infected pigs before the detection of the ASF epidemic in the country *c*, and No represents the total number of pigs in country *c*.

The value of NI was estimated as the product of four independent parameters: the probability of undetected infection in the country of origin *c* at month *m* (P_c_); the expected number of undetected outbreaks in country *c* at month *m* prior to the first notification of the disease (O_u_); the average herd size of domestic pigs in country *c* (T_c_); and the expected intra-herd ASFV prevalence (H_p_). 

The probability of undetected infection in country *c* at month *m* (P_c_) was modelled using a beta distribution with values α_1q_ and α_2q_. These values were computed as:

α_1q_ = X + 1 and α_2q_ = M − (X + 1) (3)

where X is the number of outbreaks by month in country *c* and M is the number of months considered in the analysis (i.e., number of months with information available in the OIE database from 2012 to 2020 [5]). 

The expected number of undetected outbreaks in country *c* before official notification (O_u_) was assumed to be Pert distributed. The minimum value was assumed to be 1, corresponding to the index case in country *c*. The most likely and maximum values were 1.28 and 6, respectively. These values were assumed to be the same as those estimated and published in Herrera-Ibatá et al. [14] based on the ASF outbreaks in Europe from 2007 to 2016. 

The average herd size of domestic pigs in country *c* (T_c_) was assumed normally distributed with parameters µ (mean) and σ (standard deviation), where µ is the average number of pigs and pig herds in country *c* from 2012 to 2020, and σ is the standard deviation of the number of pigs and pig herds in country *c* from 2012 to 2020 [15].

Finally, intra-herd prevalence (H_p_), representing the proportion of infected pigs in an infected (but not detected) herd [13], was modelled using a Pert distribution with minimum, most likely, and maximum values of 0.05, 0.15, and 0.32, respectively. These values were accepted to be the same as those estimated in Herrera-Ibatá et al. [14] based on the OIE data from ASF outbreaks in Europe.

#### 2.3.2. Probability of Survival of the Selected Pig (P_2_)

The probability of survival of the selected ASF-infected pig is computed as the product of two independent probabilities: the probability that a pig survives ASF infection (P_s_) and the probability that a pig survives transport from the origin country to Spain (P_t_). The probability that a pig survives ASF infection (P_s_) was modelled using a Pert distribution with a minimum, most likely, and maximum values of 0.05, 0.2, and 1. These values were based on the mortality rates of the different ASFV isolates which are responsible for the different clinical forms of the disease reported in the literature [2]. The probability that a pig survives transport and reaches a Spanish holding (P_t_) assumed a Pert distribution with minimum, most likely, and maximum values of 0.0005, 0.0027, and 0.092 based on the mortality rates during transport described by Murray and Johnson [16]. 

#### 2.3.3. Probability That an Imported ASF-Infected Pig Establishes an Effective Contact with Other Domestic Pigs in Spain, Resulting in Disease Transmission (P_3_)

The probability of establishing an effective contact that results in disease transmission (P_3_) was computed as follows:

P_3_ = P_q_ + (1 − P_q_) × P_u_ (4)

where P_q_ is the probability that pigs were not quarantined. The data available regarding biosecurity procedures for imported pigs practiced in Spanish pig farms are scarce. Therefore, the estimations published by Martínez-López et al. [17] for Spanish farms were used. P_u_ is the probability that an infected pig was not detected during quarantine. The probability of not detecting an ASF-infected pig was assumed to be the same as that used by Martínez-López et al. [17] for classical swine fever. This was based on the assumption that the clinical signs of classical swine fever are similar to those of ASF [13]. 

### 2.4. Sensitivity Analysis

Sensitivity analysis was performed in two steps to evaluate the influence of changes in input values on the model outcomes. Firstly, regression coefficients (β_i_) were calculated between each input and the annual probability of ASFV introduction into Spain. Afterwards, the inputs that were most likely to influence the final results (βi ≥ 0.1) were further analysed using the advanced sensitivity analysis tool of @RISK 8.2 by changing their base values in eight consecutive steps from a minimum of a 50% reduction to a maximum of a 50% increase. The results are reported in graphs.

## 3. Results

### 3.1. Probability of Having at Least One ASF Outbreak in Spain Due to the Import of Live Pigs

The overall mean annual probability of ASFV introduction into Spain by the legal import of live pigs was estimated as 1.07 × 10^−4^ (2.44 × 10^−4^ ~ 4.61 × 10^−6^ 95% confidence interval, CI). This value approximately corresponds to one outbreak every 9345 years if conditions remain constant. 

**Figure 2:**
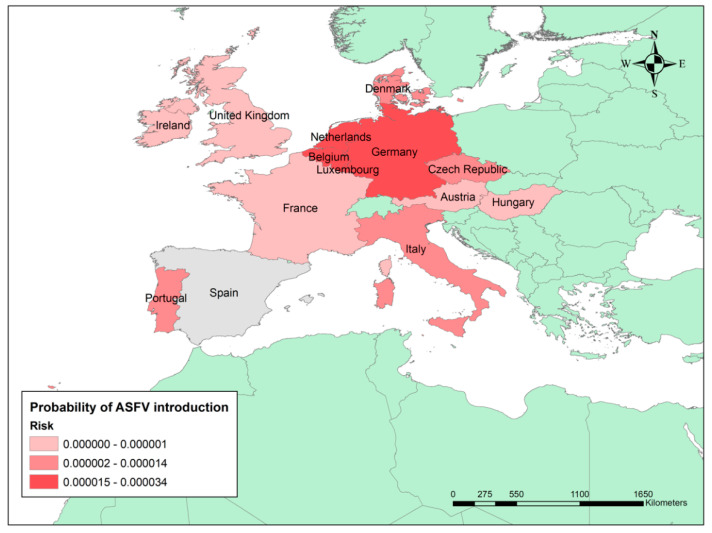
Probability of ASFV introduction into Spain by legal import of live pigs. The graduated colour map represents the final risk from the highest (darker) to the lowest (lighter) risk based on quantile classification.

The countries of origin with the highest risk were Germany (3.39 × 10^−5^), the Netherlands (1.90 × 10^−5^), Belgium (1.58 × 10^−5^), Luxembourg (1.53 × 10^−5^), and Portugal (1.49 × 10^−5^). Actually, these countries represent 92% of the overall annual risk of ASFV introduction into Spain (see Figure 2).

Analysing the temporal risk distribution, April (2.64 × 10^−5^) and February (1.75 × 10^−5^) are the months that pose the greatest risk, followed by October (1.26 × 10^−5^), March (1.21 × 10^−5^), and November (1.01 × 10^−5^).

### 3.2. Sensitivity Analysis

**Figure 3:**
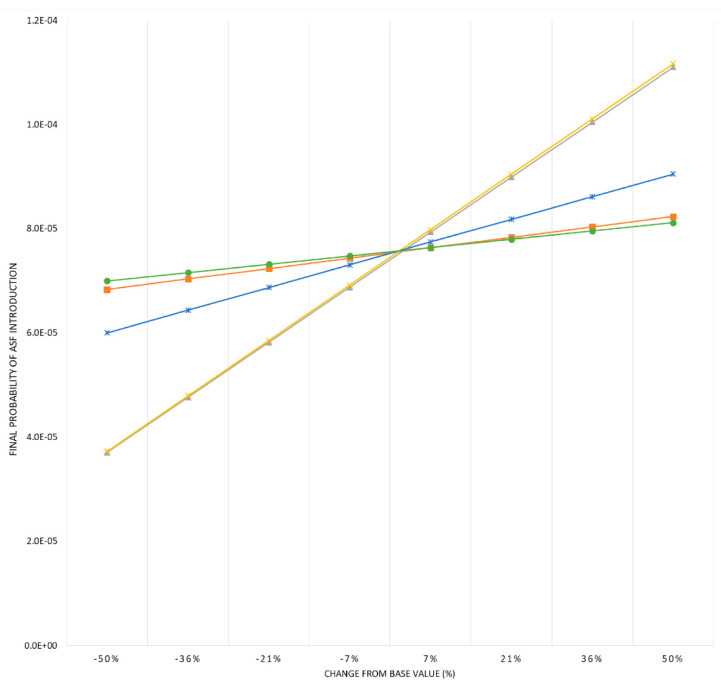
Advanced sensitivity analysis. Graph represents variations in the overall probability of ASF introduction into Spain by legal import of live pigs as a consequence of changes in the parameters with the strongest influence on the model: probability of selecting an ASF-infected pig from Portugal (■, orange colour), Germany (**✱**, blue colour), and Belgium (●, green colour); probability of surviving ASF infection (▲, grey colour) and probability of surviving transport (**𝗫**, yellow colour).

The first step of the sensitivity analysis allowed the selection of five input parameters with the strongest influence on model outcomes (βi ≥ 0.1): probability of selecting an ASF-infected pig from Portugal, Germany, and Belgium; probability of surviving ASF infection; and probability of surviving transport. The advanced sensitivity analysis revealed that model results were only substantially influenced by two of the parameters, the probability of surviving the infection and the transport. The other parameters did not have a noticeable impact on model outcomes (see Figure 3).

## 4. Discussion

The main purpose of an import risk analysis is to provide importing countries with an objective and defensible method for assessing the risk related to the importation of animals and other animal products [18]. In this study, a quantitative risk assessment was developed following the OIE guidelines in order to estimate the risk of ASF introduction into Spain via the legal import of live pigs [19]. Transparency is also fundamental to providing countries with clear and documented conclusions [18]. The model outputs obtained depend on the validity and accuracy of the information and assumptions used to parameterize the model. Accordingly, only high-quality databases and fully referenced information were used in the analysis. Notwithstanding, certain limitations may have influenced the model outcomes. For example, data from EUROSTAT used in the model depend on the inclination and capacity of countries to collect and report precise information. Therefore, a failure in these reports would result in an overestimation or underestimation of the risk. Despite this, the authors deeply assessed the results, also considering similar studies conducted [13,14,17], and concluded that the assumptions and limitations have not translated into biased estimates for the risk of ASF introduction into Spain. Additionally, it should be stressed that this is a dynamic set of information, so the risk assessment presented here should be periodically revised and updated.

The risk of introduction of ASF into Spain via the legal import of live pigs was significantly low (1.07 × 10^−4^), despite the fact that the import of live animals and their products is considered one of the most important risk factors for the introduction of ASF into an ASF-free country [6,7,8,9]. This seems to indicate that further analyses of other entry pathways, such as the importation of pig meat and biological products (semen, ova, and embryos), should also be performed. It is also important to mention that this analysis has not taken into account illegal trade, including the dispatch of feral pigs (e.g., wild boar) in EU countries [20], due to the lack of available and accredited sources of data about illicit trade of domestic and wild pigs. 

As expected, the risk of ASF introduction was concentrated in those countries with which Spain has more trade relations: Germany, the Netherlands, Portugal, Luxembourg, and Belgium. Germany was estimated to be the country with the highest contribution to the risk of ASF introduction into Spain. This was to be expected because Germany has been affected by ASF in wild boar and the domestic swine populations since September 2020 [5]. Belgium has experimented focal introductions of ASFV since 2018 but only affecting the wild boar population. This might have facilitated the eradication of the infection, with Belgium being declared ASF-free in 2020 [21]. Geographically, countries neighbouring Germany and Belgium, such as Luxembourg and the Netherlands, also pose a major risk of introduction, due to their trading relationship with Spain. In fact, the progressive increase in the import of Dutch piglets to Spain is noteworthy [12]. Furthermore, the model indicates Portugal as a country that poses a high risk, despite not being affected and being quite distant from the affected countries. This finding partly reflects the great flow of the pig trade between Spain and Portugal, given that the risk is certainly proportional to the number of animals imported annually. That risk can be also proportional to other factors such as pig population size or historical reports from previous ASF outbreaks in the country of origin. 

April and February were found to be the months with the highest risk for ASF introduction into Spain via the legal import of live pigs. This model outcome contrasts with that obtained by Mur et al. [13], where the months with highest risk were November and December. This could be partly explained by the time difference between both studies and by the use of a different database: the Trade Control and Expert System database (TRACES) versus the EUROSTAT database. 

The results of the sensitivity analysis identified the probabilities that an ASF-infected pig survives the infection and the transport as the parameters with the strongest influence on the model. Therefore, low- or medium-virulent ASF isolates may increase the introduction risk. The aforementioned isolates produce a chronic form of ASF characterized by a slower progression of the disease, milder clinical signs, and lower mortality rates [2], thereby facilitating that the ASF-infected pig survives the transport and reaches a holding in the country of destination. The situation may be aggravated by using non-authorised vaccines in attempts to stop the disease from spreading. These practices have led to the circulation of low-virulence strains in China and have facilitated the presence of animals with chronic forms and persistent infections [22,23]. Far from helping, this situation is posing a great obstacle to the early detection of the disease and its subsequent control and eradication [24]. The emergence of the aforementioned less virulent viruses increases the relevance of biosecurity measures in destination countries, such as appropriate quarantines of the live pigs imported for the early detection of chronic forms of ASF.

To the best of our knowledge, this is the first quantitative risk assessment for the risk of introducing ASF via the legal importation of live pigs specifically focused on Spain, which is the leading pig producer in the EU, together with Germany, and also in the world after China and the United States. The model presented here not only estimates the overall risk of ASF introduction into Spain but also identifies geographical areas and time periods of increased risk of ASF introduction. The epidemiological information presented herein could be used to develop and improve prevention and control strategies and, ultimately, would help reduce the risk of ASF introduction into Spain. To improve the model, other pathways of introduction such as the legal import of pork and other pig products are currently being analysed.

## 5. Conclusions

The present quantitative risk assessment was developed to estimate the risk of ASF introduction into Spain by the legal trade of live pigs. The model suggests that the legal trade of live pigs does not pose a high risk if conditions remain constant. The epidemiological information obtained could be useful to allocate human and financial resources to those geographical areas and periods with increased risk, which would contribute to improving prevention and control strategies, ultimately reducing the risk of ASF introduction into Spain.

## Figures and Tables

**Table 1:** Description and parameterization of model inputs for the risk assessment of ASF introduction into Spain by legal import of live pigs.

Notation	Definition	Parametrization	Source	Values
P_1_	Probability of selecting an ASF-infected pig from the country of origin (*c*) at month (*m*) before detection of ASFV infection	Beta (α_1_,α_2_) α_1p_ = NI + 1 α_2p_ = N_o_ – (NI + 1)	NI = P_c_ × O_u_ × T_c_ × H_p_ N_o_ = pig population in *c*	
P_c_	Probability of undetected infection in country *c*	Beta (α1,α2) α_1q_ = X + 1 α_2q_ = M – (X + 1)	OIE-WAHIS [5] X: Number of months with at least one undetected ASF outbreak in *c* M: Number of months considered in the analysis	
O_u_	Expected number of undetected outbreaks before official notification	Pert (min, most likely, max)	Herrera-Ibatá et al. [14]	Pert (1, 1.28, 6)
T_c_	Average herd size in country *c*	Normal = N_o_/S_o_		
N_o_	Pig population size in country *c*	Normal (µ,σ)	FAOSTAT (Statistical Database of the Food and Agriculture Organization of the United Nations) [15]	
S_o_	Number of pig farms in country *c*	Normal (µ,σ)	FAOSTAT [15]	
H_p_	Intra-herd prevalence	Pert (min, most likely, max)	Herrera-Ibatá et al. [14]	Pert (0.05, 0.15, 0.32)
P_2_	Probability of survival of the selected ASF-infected pig		P_s_ × P_t_	
Ps	Probability that an ASF-infected pig survives the ASF infection	Pert (min, most likely, max)	Sánchez-Vizcaíno et al. [2]	Pert (0.05, 0.2, 0,1)
Pt	Probability that an ASF-infected pig survives transportation	Pert (min, most likely, max)	Murray and Johnson [16]	Pert (0.0005, 0.0027, 0.092)
P_3_	Probability that an imported ASF-infected pig establishes an effective contact with other domestic pigs in Spain, resulting in disease transmission		P_q_ + [(1 − P_q_) × P_u_]	
P_q_	Probability that imported pigs were not quarantined	Beta (α_1_,α_2_)	Martínez-López et al. [17]	Beta (130.7, 15.4)
P_u_	Probability that an ASF-infected pig was not detected during quarantine	Beta (α_1_,α_2_)	Martínez-López et al. [17]	Beta (1.3, 34.2)
n_cm_	Import of live pigs from country *c* to Spain during month *m* (2012–2020)	Normal (µ,σ)	EUROSTAT [12]	
p_cm_	Probability that an ASF-infected pig from country *c* was introduced into a Spanish farm during month *m* and effectively contacted a susceptible pig	Binomial (n, p)	n = n_cm_ p = P_1_× P_2_ × P_3_	

## Data Availability

Not applicable.
